# Anticancer drug clustering in lung cancer based on gene expression profiles and sensitivity database

**DOI:** 10.1186/1471-2407-6-174

**Published:** 2006-06-30

**Authors:** Akihiko Gemma, Cai Li, Yuka Sugiyama, Kuniko Matsuda, Yoko Seike, Seiji Kosaihira, Yuji Minegishi, Rintaro Noro, Michiya Nara, Masahiro Seike, Akinobu Yoshimura, Aki Shionoya, Akiko Kawakami, Naoki Ogawa, Haruka Uesaka, Shoji Kudoh

**Affiliations:** 1 The Fourth Department of Medicine, Nippon Medical School, 1-1-5, Sendagi, Bunkyo-ku, Tokyo, 113–8602, Japan; 2 Genetic Lab Co., Ltd, 2–12, N27-W6, Kita-ku, Sapporo, Hokkaido, 001–0027, Japan; 3 MediBic, Daido Kasumigaseki Bldg, 8F, 1-4-2 Kasumigaseki, Chiyoda-ku, Tokyo, 100–0013, Japan

## Abstract

**background:**

The effect of current therapies in improving the survival of lung cancer patients remains far from satisfactory. It is consequently desirable to find more appropriate therapeutic opportunities based on informed insights. A molecular pharmacological analysis was undertaken to design an improved chemotherapeutic strategy for advanced lung cancer.

**Methods:**

We related the cytotoxic activity of each of commonly used anti-cancer agents (docetaxel, paclitaxel, gemcitabine, vinorelbine, 5-FU, SN38, cisplatin (CDDP), and carboplatin (CBDCA)) to corresponding expression pattern in each of the cell lines using a modified NCI program.

**Results:**

We performed gene expression analysis in lung cancer cell lines using cDNA filter and high-density oligonucleotide arrays. We also examined the sensitivity of these cell lines to these drugs via MTT assay. To obtain our reproducible gene-drug sensitivity correlation data, we separately analyzed two sets of lung cancer cell lines, namely 10 and 19. In our gene-drug correlation analyses, gemcitabine consistently belonged to an isolated cluster in a reproducible fashion. On the other hand, docetaxel, paclitaxel, 5-FU, SN-38, CBDCA and CDDP were gathered together into one large cluster.

**Conclusion:**

These results suggest that chemotherapy regimens including gemcitabine should be evaluated in second-line chemotherapy in cases where the first-line chemotherapy did not include this drug. Gene expression-drug sensitivity correlations, as provided by the NCI program, may yield improved therapeutic options for treatment of specific tumor types.

## Background

While various anti-cancer drugs have been developed, many patients with solid tumors still exhibit poor prognosis. Accordingly, it is now important to determine the appropriate use of such drugs clinically. With respect to treatment of lung cancer, there are many anti-cancer agents in use, such as cisplatin (CDDP), carboplatin (CBDCA), docetaxel, paclitaxel, vinorelbine, gemicitabine, 5-fluorouracil (5-FU), CPT-11, etc. A number of combination therapy regimens employing platinum compounds have proven to be effective[1] and are widely applied to initial treatment for unresected non-small cell lung cancer (NSCLC)[2]. In addition, docetaxel and pemetrexed have been reported to be effective in the context of second-line chemotherapy for NSCLC[3,4]. However, at present, the effect of these therapies on improving patient survival remains far from satisfactory [1-3]. It is consequently desirable to find more appropriate therapeutic opportunities based on informed insights. With the recent near-completion of the human genome sequence, genome-wide gene expression profiling through both cDNA and oligonucleotide arrays has been greatly facilitated [5-7]. There are many reports associated with isolation of molecules involved in drug sensitivity [8-10]. Of particular relevance was the use of DNA array-based methodology by the National Cancer Institute (NCI) to assess the gene expression profiles of 60 human cancer cell lines of diverse tissue origin (NCI60 set), with a view to determining associations with the extensive drug sensitivity data accumulated on this cell line cohort so far[11]. The NCI60 gene expression study was analogous in some respects to assessment of clinical tumors for markers that predict sensitivity to therapy. The essential aim of this study was to utilize similar advanced gene expression profiling technologies and drug sensitivity assays to aid in the selection of appropriate drug combinations for the treatment of lung cancer. We performed gene expression analysis in lung cancer cell lines using cDNA filter and high-density oligonucleotide arrays. We also examined the sensitivity of these cell lines to commonly used anti-cancer agents (docetaxel, paclitaxel, gemcitabine, vinorelbine, 5-FU, SN38, cisplatin (CDDP), and carboplatin (CBDCA)) via MTT assay. We related the cytotoxic activity of each of these agents to the corresponding expression pattern in each of the cell lines using a modified NCI program. To obtain our reproducible gene-drug sensitivity correlation data, we separately analyzed two sets of lung cancer cell lines, namely 10 and 19.

## Methods

### Clustering on the basis of drug activity and gene expression patterns

#### Cell lines

We analyzed the expression profiles and sensitivity to anti-cancer drugs of separate two sets of lung cancer cell lines. The first set consisted of PC9, PC7, PC14, A549, Lu65, LK2, H69, N231, Lu135, and SBC3 (Set 1). The second consisted of RERF-LC-KJ, RERF-LC-MS, RERF-LC-AI, PC1, PC3, PC6, PC10, Lu130, Lu139, Lu165, ABC-1, EBC-1, LC2/ad, LC1/sq, LC-1F, SQ5, QG56, MS-1, and SBC5 (Set 2). The PC1, PC3, PC6, PC7, PC9, PC10, PC14, and QG56 cell lines were obtained from IBL (Gumma, Japan). The A549, NCI-H69, and NCI-N231 cell lines were obtained from the American Type Culture Collection (Rockville, MD)[12]. The Lu65 and Lu135 cell lines were provided by Y. Shimosato and T. Terasaki (National Cancer Center Research Institute, Tokyo, Japan)[12]. The LK-2 and SBC-3 cell lines were obtained from the Health Science Research Resources Bank (Osaka, Japan). PC1, PC3, PC6, PC10, Lu130, Lu139, and Lu165 cell lines were provided by S. Hirohashi (National Cancer Center Research Institute, Tokyo, Japan). RERF-LC-KJ, LC2/ad, SQ5, LC1/sq, LC-1F, RERF-LC-AI, and MS-1 cell lines were obtained from the RIKEN Cell Bank (Ibaraki, Japan). RERF-LC-MS, EBC-1, SBC5, and ABC-1 cell lines were purchased from the Health Science Research Resources Bank (Osaka, Japan). PC7, PC9, PC14, A549, Lu65, RERF-LC-KJ, RERF-LC-MS, PC3, ABC-1, and LC2/ad are adenocarcinoma cell lines. LK-2, RERF-LC-AI, PC1, PC10, EBC-1, LC1/sq, LC-1F, SQ5, and QG56 are squamous cell cancer cell lines. NCI-H69, NCI-N231, Lu135, SBC-3, PC6, Lu130, Lu139, Lu165, MS-1, and SBC5 are small cell lung cancer cell lines.

#### Assay for drug activity

Estimation of cytotoxicity in the above-mentioned cell types was mediated by a rapid colorimetric assay for mitochondrial dehydrogenase activity, as previously described[13]. Briefly, cells were seeded into 12-well plates (Falcon, Lincoln Park, NJ). Following 24 hr exposure to particular anti-cancer agents, the cells were washed twice and incubated for a further 24 hr in drug-free medium. Subsequently, the cells were incubated with 0.5 mg/mL 3-(4,5-dimethylthiazol-2-yl)-2,5-diphenyl-tetrazolium bromide (MTT) for 4 hr. The blue formazan crystals, formed by viable cells, were solubilized by the addition of 10% n-dodecylsulfate sodium salt (SDS) in 0.01N HCl followed by overnight incubation. Samples were then subjected to spectrophotometric analysis at 560 nm (Ultraspec 4050; LKB, Bromma, Sweden).

#### RNA isolation, cDNA array hybridization and analysis of hybridization signals

Total RNA was isolated from each cell line using standard protocols described previously[14,15]. To avoid variations due to cell culture conditions, we cultured each untreated cell line separately in 6 different flasks. mRNA was then purified from total RNA by incubation with oligo-dT-magnetic beads (Toyobo Co., Osaka, Japan)[16]. The ElectorGene Array System (GeneticLab. Co., Ltd. Sapporo, Japan) was used for filter-based cDNA array analysis, as previously reported[16]. Thirteen hundred species of human DNA fragments are spotted in duplicate on a filter. The genes represented on this filter included cancer-related and drug resistance-associated genes, as well as housekeeping genes and non-mammalian genes as negative controls. To prepare the probes, reverse transcription was performed using Reverse Transcriptase, ReverTraAce (Toyobo Co., Osaka, Japan), together with a random 9 mer (Toyobo Co., Osaka, Japan) as the primer and 5 μg of polyA RNA. The probes were labelled with biotin by incorporation of biotin-16-deoxyuracil triphosphate (dUTP) during the synthesis of cDNA. The filters were pre-incubated in 20 ml of PerfectHyb (Toyobo Co., Osaka, Japan) at 68°C for 30 min. The biotin-labeled probes were denatured and added to the pre-hybridization solution. The filters were incubated overnight at 68°C in the hybridization mixture. After washing, specific signals on the filters were detected by the Imaging High – Chemilumi – Detection kit (Toyobo Co., Osaka, Japan). CDP-Star substrate (Tropix, Bedford, MA) was used as the chemiluminescence substrate. A chemiluminescence image of the filter was acquired by Fluor-S (Bio-Rad, Hercules, CA). The gene expression images were quantified by measuring the intensity of the signals using Imagene (BioDiscovery, Los Angeles, CA). The signal intensity among filters was analyzed by ElectorGene Finding System (GeneticLab, Sapporo, Japan). The background threshold was set at a level of 3-fold higher than the negative control. Signal intensities were normalized by comparison with the average values of all probe. We also performed high-density oligonucleotide array analysis using Affymetrix GeneChip technology (Affymetrix, Santa Clara, CA). This oligonucleotide microarray contains 22,282 transcripts (HG-U133A, Affymetrix, Santa Clara, CA). Total RNA was used to synthesize double-strand cDNA with ReverTraAce and a T7-(dT)24 primer (Metabion, Germany). Then, biotinylated cRNA was synthesized from the double-stranded cDNA using the RNA Transcript Labeling kit (Enzo Life Sciences, Farmingdale, NY) and was purified and fragmented. The fragmented cRNA was hybridized to the oligonucleotide microarray, which was washed and stained with streptavidine-phycoerythrin. Scanning was performed with an Agilent Microarray Scanner (Agilent Technologies, Palo Alto, CA). GeneChip analysis was performed based on the Affymetrix GeneChip Mannual (Affymetrix Inc., Santa Clara, CA) with Microarray Analysis Suite (MAS) 5.0, Data Mining Tool (DMT) 2.0, and Microarray Database software. The data we generated by GeneChip was deposited in Gene Expression Omnibus (GEO)(GEO accession: GSE4127)(17).

#### Data analysis

We performed data cleansing for filter arrays as follows. Firstly, the gene expression matrix [T] was scaled by using the average of all probe sets. Each of the filter arrays contained three spots of negative control (pUC), so we figured out their average signal value *M*. We defined 3 *M *as the threshold value, and transformed the numerical signal values < 3 M to "Nan" (not a number). After omitting the rows holding "Nan" more than one, we selected 600 genes for this analysis. Data analysis for the correlation coefficients that related the drug activity patterns to the expression patterns of the genes was principally performed by a modified NCI program[11]. The symbol [A] (GI_50_) refers to the drug activity matrix in which the rows represent the anti-cancer drugs and the columns represent the human lung cancer cell lines. The symbol [T] (gene expression) refers to the gene expression matrix in which the rows represent individual genes and the columns represent the cell lines. In order to analyze the relationship between gene expression and drug activity, we generated the gene-drug correlation matrix [AT] (correlation coefficient) in which the rows represent the genes and the columns represent the drugs. Firstly, we subtracted its mean value from the matrix [A] in the direction of row and columns for a pre-treatment. Secondly, we normalized each element in the matrix [A] by subtracting its row-wise mean and dividing by its row-wise standard deviation; normalized [T] was generated in a similar way. Finally, we took the inner product of the matrix [A] and the transpose of the matrix [T]. The resulting matrix [AT] implied the Pearson correlation coefficients (1) that reflected the relationship between drug activity and gene expression.

(1) The Pearson correlation coefficient r is given by the formula

r=1n−1∑k=1n(Ak−A¯ΦA)(Tk−T¯ΦT)=1n−1∑k=1n(Ak−A¯)(Tk−T¯)1n−1∑k=1n(Ak−A¯)21n−1∑k=1n(Tk−T¯)2ΦX:the standard deviation of XX¯:the mean of X
 MathType@MTEF@5@5@+=feaafiart1ev1aaatCvAUfKttLearuWrP9MDH5MBPbIqV92AaeXatLxBI9gBaebbnrfifHhDYfgasaacH8akY=wiFfYdH8Gipec8Eeeu0xXdbba9frFj0=OqFfea0dXdd9vqai=hGuQ8kuc9pgc9s8qqaq=dirpe0xb9q8qiLsFr0=vr0=vr0dc8meaabaqaciaacaGaaeqabaqabeGadaaakqaabeqaauaabmqaceaaaeaacqWGYbGCcqGH9aqpdaWcaaqaaiabigdaXaqaaiabd6gaUjabgkHiTiabigdaXaaadaaeWbqaaiabcIcaOmaalaaabaGaemyqae0aaSbaaSqaaiabdUgaRbqabaGccqGHsislcuWGbbqqgaqeaaqaaGGaaiab=z6agnaaBaaaleaacqWGbbqqaeqaaaaaaeaacqWGRbWAcqGH9aqpcqaIXaqmaeaacqWGUbGBa0GaeyyeIuoakiabcMcaPiabcIcaOmaalaaabaGaemivaq1aaSbaaSqaaiabdUgaRbqabaGccqGHsislcuWGubavgaqeaaqaaiab=z6agnaaBaaaleaacqWGubavaeqaaaaakiabcMcaPaqaaiabg2da9maalaaabaWaaSaaaeaacqaIXaqmaeaacqWGUbGBcqGHsislcqaIXaqmaaWaaabCaeaacqGGOaakcqWGbbqqdaWgaaWcbaGaem4AaSgabeaakiabgkHiTiqbdgeabzaaraGaeiykaKIaeiikaGIaemivaq1aaSbaaSqaaiabdUgaRbqabaGccqGHsislcuWGubavgaqeaiabcMcaPaWcbaGaem4AaSMaeyypa0JaeGymaedabaGaemOBa4ganiabggHiLdaakeaadaGcaaqaamaalaaabaGaeGymaedabaGaemOBa4MaeyOeI0IaeGymaedaamaaqahabaGaeiikaGIaemyqae0aaSbaaSqaaiabdUgaRbqabaGccqGHsislcuWGbbqqgaqeaiabcMcaPmaaCaaaleqabaGaeGOmaidaaaqaaiabdUgaRjabg2da9iabigdaXaqaaiabd6gaUbqdcqGHris5aaWcbeaakmaakaaabaWaaSaaaeaacqaIXaqmaeaacqWGUbGBcqGHsislcqaIXaqmaaWaaabCaeaadaqadaqaaiabdsfaunaaBaaaleaacqWGRbWAaeqaaOGaeyOeI0IafmivaqLbaebaaiaawIcacaGLPaaadaahaaWcbeqaaiabikdaYaaaaeaacqWGRbWAcqGH9aqpcqaIXaqmaeaacqWGUbGBa0GaeyyeIuoaaSqabaaaaaaaaOqaauaabmqaceaaaeaacqqHMoGrdaWgaaWcbaGaemiwaGfabeaakiabcQda6iabbsha0jabbIgaOjabbwgaLjabbccaGiabbohaZjabbsha0jabbggaHjabb6gaUjabbsgaKjabbggaHjabbkhaYjabbsgaKjabbccaGiabbsgaKjabbwgaLjabbAha2jabbMgaPjabbggaHjabbsha0jabbMgaPjabb+gaVjabb6gaUjabbccaGiabb+gaVjabbAgaMjabbccaGiabbIfaybqaaiqbdIfayzaaraGaeiOoaOJaeeiDaqNaeeiAaGMaeeyzauMaeeiiaaIaeeyBa0MaeeyzauMaeeyyaeMaeeOBa4MaeeiiaaIaee4Ba8MaeeOzayMaeeiiaaIaeeiwaGfaaaaaaa@C4B0@

Hierarchical clustering helps to comprehend a characteristic of huge volumes of data. With cluster analysis, the elements are divided into groups that show similar patterns by calculating the distances between their respective rows and columns. The AT-clustered image map (CIM), indicating the correlation coefficients between gene expression and drug sensitivity in the 10 human lung cancer cell lines, was obtained by the linkage-average clustering method, also known as UPGMA (un-weighted pair-group method using arithmetic average). The statistical algorithms and the graphical outputs described here were implemented in MATLAB 6.5 Release 13 (the MathWorks, Inc., US).

## Results

### Clustering on the basis of drug activity and gene expression patterns

**Figure 1:**
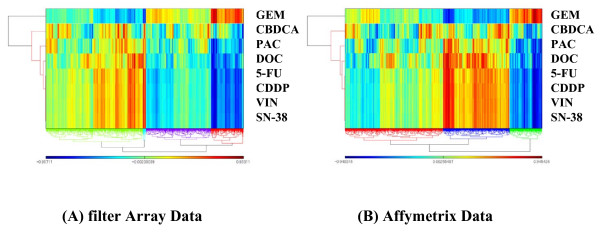
Clustered image map (CIM) relating drug activity to gene expression in lung cancer cell lines – Experiment 1. (A) using filter array, (B) using GeneChip. The cluster trees of drugs (y axis) and gene expression (x axis) were shown in the CIM. Each block of colors represents correlations between clusters of genes and drugs; red (high positive correlation) and blue (negative correlation). In this analysis, gemcitabine belonged to an isolated cluster (Fig. 1A,B). GEM; gemcitabine, PAC; paclitaxel, DOC; docetaxel, VIN; vinorelbine

**Figure 2:**
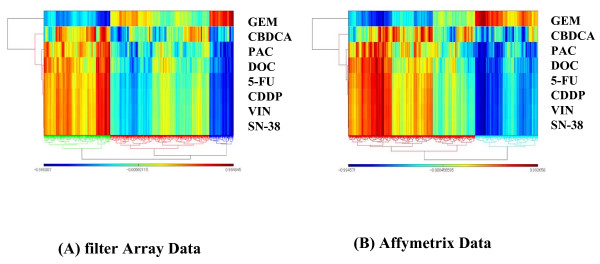
Clustered image map (CIM) relating drug activity to gene expression in NSCLC cell lines – Experiment 1. (A) using filter array, (B) using GeneChip. The cluster trees of drugs (y axis) and gene expression (x axis) were shown in the CIM. Each block of colors represents correlations between clusters of genes and drugs; red (high positive correlation) and blue (negative correlation). In this gemcitabine belonged to an isolated cluster (Fig. 2A,B). GEM; gemcitabine, PAC; paclitaxel, DOC; docetaxel, VIN; vinorelbine

**Figure 3:**
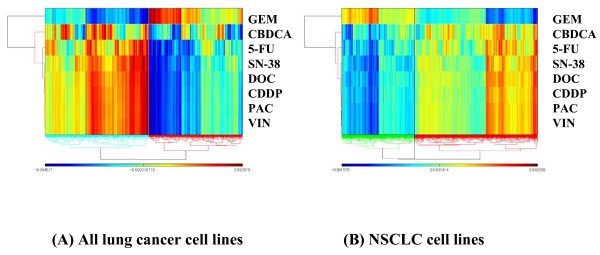
Clustered image map (CIM) relating drug activity to gene expression in lung cancer cell lines using GeneChip – Experiment 2. (A) all lung cancer cell lines, (B) NSCLC. The cluster trees of drugs (y axis) and gene expression (x axis) were shown in the CIM. Each block of colors represents correlations between clusters of genes and drugs; red (high positive correlation) and blue (negative correlation). In this analysis, gemcitabine belonged to an isolated cluster (Fig. 3A,B). GEM; gemcitabine, PAC; paclitaxel, DOC; docetaxel, VIN; vinorelbine

We used filter-based DNA arrays, representing 1,302 cancer-related and drug resistance-associated genes, and Affymetrix GeneChip technology to perform gene expression profile analysis of 10 human cancer cell lines. To avoid the influence of cell culture conditions, we separately cultured each cell line in 6 bottles[16]. The controls including GAPDH, β-actin genes, were located in duplicate at the outer line in the opposite angle. A standard curve was obtained by the calculation of serial diluted spots of GAPDH. The expression level of each gene was calculated by comparison with the internal standard. Drug sensitivity tests, namely by MTT analysis, were performed on the 10 lung cancer cell lines. Eight anti-cancer drugs currently used for lung cancer chemotherapy; docetaxel, paclitaxel, gemcitabine, vinorelbine, 5-FU, SN38, CDDP, and CBDCA, were selected for our analyses. Table 1 shows the growth inhibitory activities (GI50) levels of these anti-cancer agents against the lung cancer cell lines. We then analyzed the gene expression profiling data in relation to the activity profiles of the 8 drugs examined. The drugs were clustered on the basis of Pearson correlation coefficients that related their activity patterns across the 10 cell lines to the expression pattern of the genes in the cell lines[11]. The AT-matrix clustered image map (CIM) summarized the relationship between drug sensitivity and gene expression, as it allows the visualization of patterns of similarity in large sets of high-dimensional data (Fig. 1B)[16]. In this analysis, gemcitabine were located in separate clusters (Fig. 1A). We performed an analogous gene expression profiling screen using Affymetrix GeneChip arrays, receiving the same results with respect to drug clusters (Fig. 1B). The results of the analysis of NSCLC cell lines was similar to that seen with all lung cancer cell lines (Figure 2A,B). However, it is sometimes difficult to consistently reproduce data of the gene-drug sensitivity correlation using cDNA array technique and clinical response data. To obtain reproducible data, we separately performed Affymetrix GeneChip array-based gene expression profile analyses and sensitivity tests on another set of 19 human lung cancer cell lines and examined the sensitivity of these separate sets to 8 commonly used anti-cancer agents. Table 2 shows the GI50 levels of these anti-cancer agents against the lung cancer cell lines (Set 2) [see Additional file 1 ]. The drugs were clustered using Set 2, 19 cell lines (Set 2)[11]. In this analysis, gemcitabine was again located in separate clusters (Fig. 3A). The results of the analysis of NSCLC cell lines of Set 2 was also similar (Fig. 3B). Several genes, were commonly listed that differentiated gemcitabine from the others in Sets 1 and 2. These were a LEMT1 domain containing gene (Accession No. NM_015416), a dehydrogenase gene (Accession No. AL050217), and a gene of homo sapiens hypothetical protein (Accession No. NM_016402). A LEMT1 domain containing gene was reported to contribute to neoplastic cellular transformation[18]. A dehydrogenase gene constitutes a large protein family of NAD(P)(H)-dependent oxidoreductase[19]. A gene of homo sapiens hypothetical protein is similar to a heat shock 70kDa protein 8 isoform[20]. Presently, their functions involved in drug sensitivity remains unclear.

## Discussion

Here, we used a DNA array-based gene expression profiling approach, together with assessment of the cytotoxic activity of several widely applied anti-cancer agents, in two collections of human lung cancer cell lines. In particular, we related gene expression and drug sensitivity patterns in these cell lines. According to our separate two combined cytotoxicity and transcriptomic analyses, gemcitabine belonged to an isolated cluster. These results would suggest that combination chemotherapy regimens including gemcitabine could be a candidate for initial treatment, because combinations of drugs belonging to different clusters could expand the spectrum of the chemotherapy. Gemcitabine was deemed from our studies to be a good candidate for the treatment of recurrent or refractory NSCLC. Recently, an *in silico *search was performed to identify genes whose expression was positively or negatively correlated with sensitivity to four platinum compounds (CDDP, CBDCA, oxaliplatin and tetraplatin); the publicly available databases of the National Cancer Institute (NCI)(21) were used for this purpose[22]. CDDP, CBDCA, oxaliplatin and tetraplatin are platinum-based compounds that are classically thought to have a similar spectrum of activities, allowing for one agent to be substituted for the other[23]. Important similarities were noticed between CDDP and CBDCA on one hand, and tetraplatin and oxaliplatin on the other hand[22]. The gene-drug correlations using NCI program in these study may be a valuable tool for the identification of determinants of anticancer drug activity in tumors and for the design of cancer chemotherapy.

Vekris *et al*. described several limitations to the type of study that they have developed. 1. The evaluation of gene expression was performed on a subset of 1416 genes and molecular markers. 2. The level of expression of the 1416 molecular markers was determined with a technique that was still under development and not fully validated. 3. The criterion for drug cytotoxicity that has been retained by the NCI is the 50% growth inhibitory concentration (GI50) rather than the overall number of cells killed. There were several differences between the study of Vekris *et al*. and ours, most notably with respect to the cell lines analyzed in each case. Our study focused on lung cancer cell lines, whereas Vekris *et al*. utilized available information on the NCI60 set, a wide range of cancer cell lines[22]. The evaluation of gene expression by Vekris *et al*. was performed on a subset of 1,416 genes, which represents a relatively small fraction of the total transcriptome. We analyzed gene expression using two different DNA array formats, namely spotted filter (data not shown) and genome-wide GeneGhip arrays, with similar results being obtained. In addition, we separately analyzed two sets of lung cancer cell lines, 10 and 19 lines to obtain our reproducible gene-drug sensitivity correlation data.

Using cDNA array technique and clinical response data, it is sometimes difficult to consistently reproduce gene-drug sensitivity correlation data. These data were often influenced by sampling methods, sample preservation status, tumor size, tumor environment status including tumor vessels and inflammation, etc. In the study of Vekris *et al*. and ours, these influences were small because cancer cell lines were used. However, cell lines differ from tumor cells and should therefore be considered as surrogates that may contain information on the molecular cell biology and molecular pharmacology of cancer.

In the treatment of lung cancer, a number of combination therapy regimens employing platinum compounds have proven to be effective[1] and are widely applied as first-line treatment for unresected NSCLC; for example, CDDP + docetaxel, CBDCA + paclitaxel, CDDP + gemcitabine, CDDP + CPT-11, CDDP + paclitaxel, CDDP + vinorelbine, etc[2]. In addition, docetaxel and pemetrexed have been reported to be effective in the context of second-line chemotherapy for NSCLC[3,4]. However, how were the anti-cancer agents in these reports selected? It is consequently desirable to find more appropriate therapeutic opportunities based on informed insights.

## Conclusion

The results of our molecular pharmacological analysis suggest that chemotherapy regimens including gemcitabine should be evaluated in second-line chemotherapy if the initial chemotherapy does not include this drugs. A total design approach to cancer chemotherapy through the gene-drug correlations using NCI program may yield improved therapeutic options.

## Competing interests

The author(s) declare that they have no competing interests.

## Authors' contributions

AG and SK designed this study, analyzed and interpreted the data. CL, YS, and KM carried out sensitivity test, YS, and MS carried out cDNA array analysis. SK, YM, RN, MN, and AY carried out cell culture and RNA extraction. AS, and NO carried out acquisition of cDNA array data, HU carried out statistic analysis. All authors read and approved the final manuscript.

## Pre-publication history

The pre-publication history for this paper can be accessed here:



## Supplementary Material

Additional File 1 Table 2 Growth inhibitory activities (GI50)(μg/ml) of various anti-cancer agents against 19 human lung cancer cell lines – Set 2 Click here for file

## Figures and Tables

**Table 1:** Growth inhibitory activities (GI50)(μg/ml) of various anti-cancer agents against 10 human lung cancer cell lines – Set 1

Drug										
	PC7	PC9	PC14	A549	Lu65	LK-2	H69	N231	Lu135	SBC-3
CDDP	4.55	3.53	2.86	5.46	3.39	4.72	1.64	1.25	0.22	4.84
SN38	5.05	3.45	3.79	6.80	0.48	4.25	0.77	0.66	0.05	0.07
VIN	2.64	1.43	>10	>10	0.06	0.02	0.02	0.01	0.02	0.51
DOC	2.46	21.34	20.38	21.31	3.61	26.31	4.72	6.04	0.53	>50
GEM	<0.05	14.28	>500	>500	79.53	54.61	3.73	3.88	<0.05	<0.05
5FU	2.76	5.84	>10	>10	8.76	>10	>10	>10	5.68	0.51
CBDCA	95	70	>100	46.3	71	0.225	7.54	45	61	3.5
PAC	6.7	39	34	51	34	20	<0.001	7	5.5	0.5
