# Supplementary material for: Anticancer drug clustering in lung cancer based on gene expression profiles and sensitivity database
Source: BMC Cancer. 2006 Jun 30;6:174. doi: 10.1186/1471-2407-6-174 (PMC1533844; doi:10.1186/1471-2407-6-174)
Supplement: Additional File 1 — Table 2 Growth inhibitory activities (GI50)(μg/ml) of various anti-cancer agents against 19 human lung cancer cell lines – Set 2 [file 1471-2407-6-174-S1.doc]

**Table 2. Growth inhibitory activities (GI50)(μg/ml) of various anti-cancer agents against 19 human lung cancer cell lines**

**– Set 2**

|  | PC-3 | ABC-1 | RERF-  LC-KJ | RERF-  LC-MS | LC 2  /ad | PC-1 | PC-10 | SQ-5 | LC-1  /sq | LC-1F | EBC-1 | QG-56 | RERF-  LC-AI | Lu130 | Lu139 | Lu165 | PC-6 | SBC-5 | MS-1 |
| --- | --- | --- | --- | --- | --- | --- | --- | --- | --- | --- | --- | --- | --- | --- | --- | --- | --- | --- | --- |
| CDDP | 21.3 | 2.50 | 7.50 | 3.50 | 7.5 | 11.3 | 3.00 | 5.00 | 25. | 21.3 | 4.00 | 4.00 | 2.00 | 5.00 | 4.00 | 2.50 | 4.0 | 5.00 | 17.5 |
| SN-38 | >50 | >50 | >50 | >50 | >50 | >50 | >50 | >50 | >50 | >50 | >50 | >50 | >50 | >50 | 0.03 | 0.05 | >50 | 0.30 | >50 |
| VIN | >10 | 10.00 | >10 | >10 | >10 | >10 | >10 | 10.0 | >10 | >10 | >10 | 6.30 | >10 | >10 | 0.003 | 0.50 | >10 | >10 | >10 |
| DOC | 30. | 25.00 | 35.00 | 30.00 | 35 | 7.50 | 25 | 5.00 | 30 | 8.75 | 30.00 | 20.00 | 30.00 | 30 | 10 | 5.00 | 7.5 | 20.00 | 10.0 |
| GEM | >500 | 100 | 500 | >500 | >500 | 0.05 | 5.00 | 3.00 | >500 | >500 | 0.75 | >500 | 7.50 | 0.50 | 500 | 1.00 | >500 | 0.10 | 100. |
| 5-FU | >50 | 7.50 | >50 | 50.00 | >50 | 50.0 | >50 | 8.75 | >50 | 50 | 5.00 | 7.50 | 10.00 | 10.0 | 10.0 | 2.50 | 50. | 4.50 | >50 |
| CBDCA | 100 | 75.00 | 100. | 50.00 | >100 | 75. | 50.0 | 35. | >100 | >100 | 40.00 | 45.00 | 40.00 | 8.75 | 62.5 | 30.0 | >100 | 50.00 | 100. |
| PAC | 5.0 | 8.75 | 4.00 | 5.00 | 30 | 7.50 | 20 | 2.00 | 20 | 7.50 | 5.00 | 6.30 | 4.50 | 3.00 | 2.00 | 2.50 | 3.0 | 10.00 | 3.75 |
